# Supplementary material for: Effect of Boric Acid on the Ionization Equilibrium of α-Hydroxy Carboxylic Acids and the Study of Its Applications
Source: Molecules. 2023 Jun 12;28(12):4723. doi: 10.3390/molecules28124723 (PMC10300858; doi:10.3390/molecules28124723)
Supplement: Supplementary file 1 [file molecules-28-04723-s001.zip › molecules-2408721-supplementary.pdf]

Supporting Information

(a) By fitting the experimental data in Figure 1, equations for the change in the pH with the addition of boric acid for HCA concentrations of 0.1 mol/kg were obtained:

$$y(GA) = -0.0029x^3 + 0.0196x^2 - 0.0741x + 2.4028 \quad R^2 = 0.9813, \tag{S1}$$

$$y(LA) = -0.0083x^3 + 0.0498x^2 - 0.148x + 2.27 \quad R^2 = 0.999, \tag{S2}$$

$$y(MA) = -0.0279x^3 + 0.1782x^2 - 0.428x + 2.2087 \quad R^2 = 0.9969, \tag{S3}$$

$$y(GlcA) = -0.053x^3 + 0.3054x^2 - 0.6108x + 2.4808 \quad R^2 = 0.9948, \tag{S4}$$

$$y(H_2Mi) = -0.0127x^3 + 0.0953x^2 - 0.2858x + 2.1996 \quad R^2 = 0.9964, \tag{S5}$$

$$y(L\text{-}TA) = -0.0269x^3 + 0.1735x^2 - 0.4228x + 2.0361 \quad R^2 = 0.9949, \tag{S6}$$

$$y(D\text{-}TA) = -0.0336x^3 + 0.2085x^2 - 0.4673x + 1.9962 \quad R^2 = 0.9952, \tag{S7}$$

$$y(CA) = -0.0493x^3 + 0.2996x^2 - 0.6182x + 2.0197 \quad R^2 = 0.9896, \tag{S8}$$

where y is the pH of the HCA, x is the molar ratio of boric acid to HCA, and R is the correlation coefficient.

The derivatives of Equations (S1)–(S8) provide equations for the rates of change of the pH with the addition of boric acid for HCA concentrations of 0.1 mol/kg:

$$\frac{dy(GA)}{dx} = -0.0087x^2 + 0.0392x - 0.0741, \tag{S9}$$

$$\frac{dy(LA)}{dx} = -0.0249x^2 + 0.0996x - 0.148, \tag{S10}$$

$$\frac{dy(MA)}{dx} = -0.0837x^2 + 0.3564x - 0.428, \tag{S11}$$

$$\frac{dy(GlcA)}{dx} = -0.159x^2 + 0.6108x - 0.6108 \quad , \tag{S12}$$

$$\frac{dy(H_2Mi)}{dx} = -0.0381x^2 + 0.1906x - 0.2858 \quad , \tag{S13}$$

$$\frac{dy(L-TA)}{dx} = -0.0807x^2 + 0.347x - 0.4228 \quad , \tag{S14}$$

$$\frac{dy(D-TA)}{dx} = -0.1008x^2 + 0.417x - 0.4673 \quad , \tag{S15}$$

$$\frac{dy(CA)}{dx} = -0.1479x^2 + 0.5998x - 0.6182 \quad , \tag{S16}$$

where y is the pH of the HCA, x is the molar ratio of boric acid to HCA, and $\frac{dy}{dx}$ is the rate of change of the pH with the amount of boric acid.

(b) By fitting the experimental data in Figure 2, the equations for the change in pH with the addition of boric acid at HCA concentrations of 0.2 mol/kg can be obtained:

$$\begin{aligned} &y(GA) = -0.002x^3 + 0.0212x^2 - 0.136x + 2.2781 \\ &R^2 = 0.9976 \end{aligned} \quad , \tag{S17}$$

$$\begin{aligned} &y(LA) = -0.0204x^3 + 0.1236x^2 - 0.3135x + 2.1047 \\ &R^2 = 0.9979 \end{aligned} \quad , \tag{S18}$$

$$\begin{aligned} &y(MA) = -0.0713x^3 + 0.4202x^2 - 0.8324x + 2.0258 \\ &R^2 = 0.9811 \end{aligned} \quad , \tag{S19}$$

$$\begin{aligned} &y(GlcA) = -0.0829x^3 + 0.4859x^2 - 0.9575x + 2.2724 \\ &R^2 = 0.9712 \end{aligned} \quad , \tag{S20}$$

$$\begin{aligned} &y(H_2Mi) = -0.0451x^3 + 0.2786x^2 - 0.6234x + 2.0327 \\ &R^2 = 0.9946 \end{aligned} \quad , \tag{S21}$$

$$\begin{aligned} &y(L\text{-}TA) = -0.0651x^3 + 0.3935x^2 - 0.797x + 1.8141 \\ &R^2 = 0.9794 \end{aligned} \quad , \tag{S22}$$

$$\begin{aligned} &y(D\text{-}TA) = -0.0589x^3 + 0.3386x^2 - 0.6683x + 1.7495 \\ &R^2 = 0.9576 \end{aligned} \quad , \tag{S23}$$

$$y(CA) = -0.1076x^3 + 0.6186x^2 - 1.1185x + 1.7967$$
$$R_2 = 0.9562 \quad , \qquad (S24)$$

where y is the pH of the HCA, x is the molar ratio of boric acid to HCA, and R is the correlation coefficient.

The derivatives of Equations (S17)–(S24) yield the equations for the rate of change of the pH with the addition of boric acid at a concentration of 0.2 mol/kg of HCA:

$$\frac{dy(GA)}{dx} = -0.006x^2 + 0.0424x - 0.136 \quad , \qquad (S25)$$

$$\frac{dy(LA)}{dx} = -0.0612x^2 + 0.2472x - 0.3135 \quad , \qquad (S26)$$

$$\frac{dy(MA)}{dx} = -0.2139x^2 + 0.8404x - 0.8324 \quad , \qquad (S27)$$

$$\frac{dy(GlcA)}{dx} = -0.2487x^2 + 0.9718x - 0.9575 \quad , \qquad (S28)$$

$$\frac{dy(H_2Mi)}{dx} = -0.1353x^2 + 0.5572x - 0.6234 \quad , \qquad (S29)$$

$$\frac{dy(L-TA)}{dx} = -0.1953x^2 + 0.787x - 0.797 \quad , \qquad (S30)$$

$$\frac{dy(D-TA)}{dx} = -0.1767x^2 + 0.6772x - 0.6683 \quad , \qquad (S31)$$

$$\frac{dy(CA)}{dx} = -0.3228x^2 + 1.2372x - 1.1185 \quad , \qquad (S32)$$

where y is the pH of the HCA, x is the molar ratio of boric acid to HCA, and $\frac{dy}{dx}$ is the rate of change of the pH with the amount of boric acid.

(c) By fitting the experimental data in Figure 3, equations for the changes in the pH with the addition of boric acid at an HCA concentration of 0.5 mol/kg were obtained:

$$y(GA) = -0.0926x^3 + 0.371x^2 - 0.5586x + 2.0794$$
$$R^2 = 0.9997 \quad , \qquad (S33)$$

$$y(LA) = -0.5556x^3 + 1.244x^2 - 1.098x + 1.8662$$
$$R^2 = 0.9968 \quad , \qquad (S34)$$

$$y(MA) = -1.9676x^3 + 4.2103x^2 - 2.98x + 1.8365$$
$$R^2 = 0.9887 \quad , \qquad (S35)$$

$$y(GlcA) = -2.6389x^3 + 5.4315x^2 - 3.7308x + 2.1155$$
$$R^2 = 0.9914 \quad , \qquad (S36)$$

$$y(H_2Mi) = -1.3426x^3 + 2.8532x^2 - 2.1479x + 1.7522$$
$$R^2 = 0.9942 \quad , \qquad (S37)$$

$$y(L\text{-}TA) = -1.7361x^3 + 3.5952x^2 - 2.5567x + 1.601$$
$$R^2 = 0.9938 \quad , \qquad (S38)$$

$$y(D\text{-}TA) = -1.7245x^3 + 3.5288x^2 - 2.4927x + 1.5632$$
$$R^2 = 0.9964 \quad , \qquad (S39)$$

$$y(CA) = -1.7245x^3 + 4.0556x^2 - 3.0481x + 1.601$$
$$R^2 = 0.995 \quad , \qquad (S40)$$

where y is the pH of the HCA, x is the molar ratio of boric acid to HCA, and R is the correlation coefficient.

The derivatives of Equations (S33)–(S40) provide equations for the rates of change of the pH with the addition of boric acid at a concentration of 0.5 mol/kg of HCA:

$$\frac{dy(GA)}{dx} = -0.2778x^2 + 0.742x - 0.5586 \quad , \qquad (S41)$$

$$\frac{dy(LA)}{dx} = -1.6668x^2 + 2.488x - 1.098 \quad , \qquad (S42)$$

$$\frac{dy(MA)}{dx} = -5.9028x^2 + 8.4206x - 2.98 \quad , \qquad (S43)$$

$$\frac{dy(GlcA)}{dx} = -7.9167x^2 + 10.827x - 3.7308 \quad , \qquad (S44)$$

$$\frac{dy(H_2Mi)}{dx} = -4.0278x^2 + 5.7064x - 2.1479 \quad , \qquad (S45)$$

$$\frac{dy(L-TA)}{dx} = -5.2083x^2 + 7.1904x - 2.5567 \quad , \qquad (S46)$$

$$\frac{dy(D-TA)}{dx}=-5.1735x^2+7.0576x-2.4927, \quad (S47)$$

$$\frac{dy(CA)}{dx}=-5.1735x^2+8.112x-3.0481 \quad (S48)$$

where y is the pH of the HCA, x is the molar ratio of boric acid to HCA, and $\frac{dy}{dx}$ is the rate of change of the pH with the amount of boric acid.

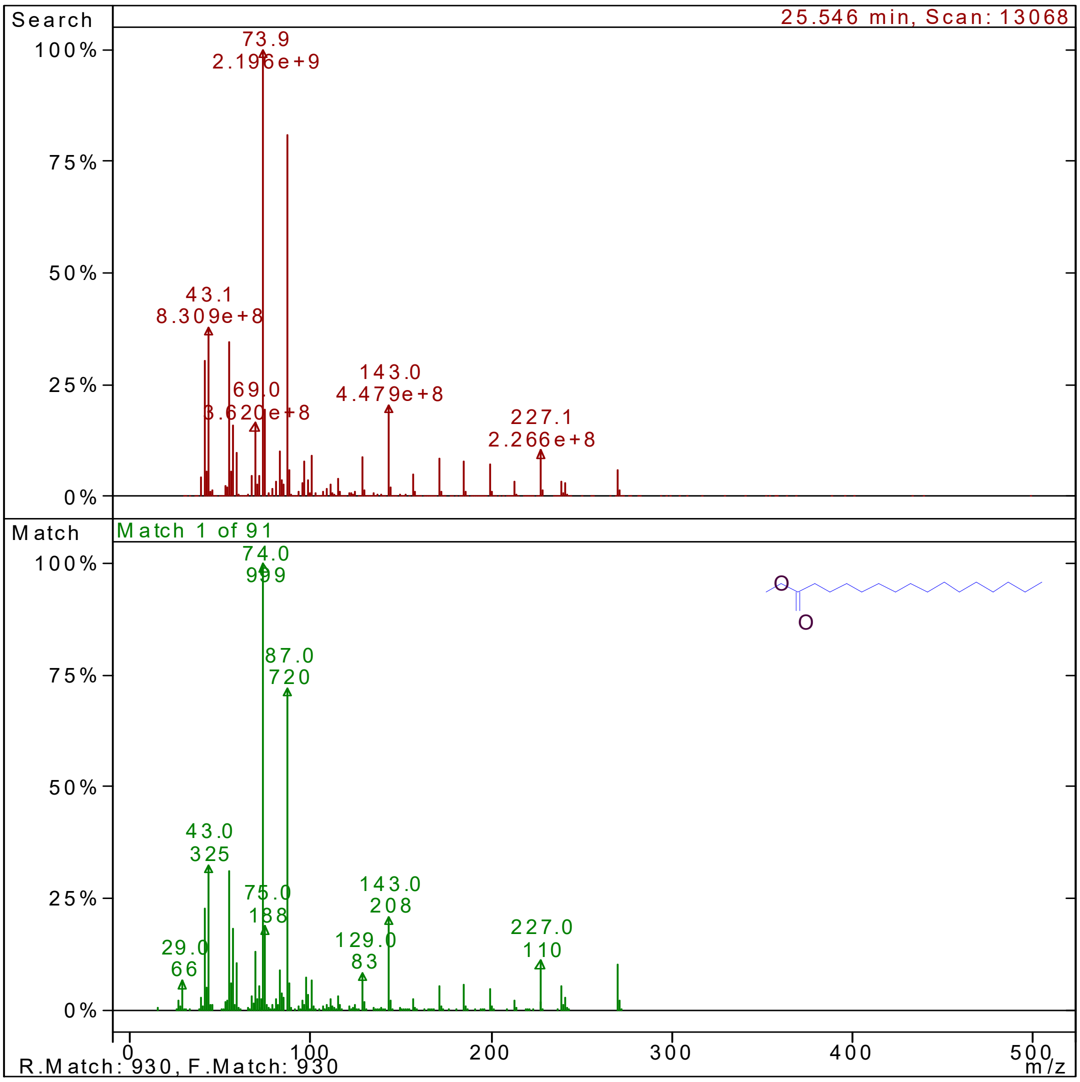

Figure S1. Mass spectrum of methyl palmitate.

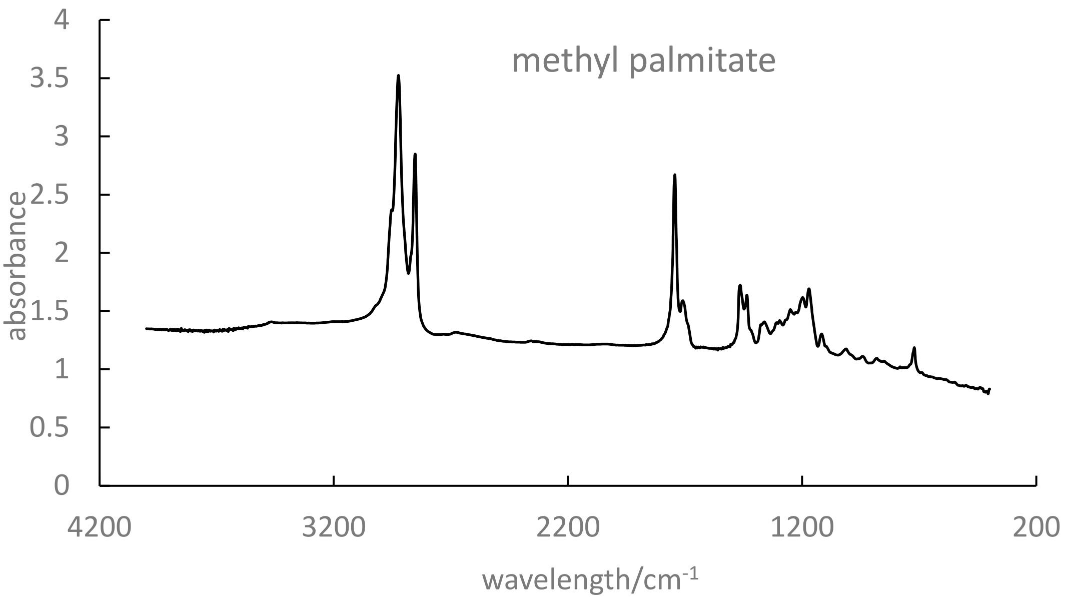

Figure S2. Infrared spectrum of methyl palmitate.
